# The three ‘S’s of editing: story, structure, and style

**DOI:** 10.1007/s40037-016-0284-2

**Published:** 2016-07-26

**Authors:** Chris Watling

**Affiliations:** Office of Postgraduate Medical Education, Schulich School of Medicine and Dentistry, Western University, London, Ontario Canada

In the writer’s craft section we offer simple tips to improve your writing in one of three areas: Energy, Clarity and Persuasiveness. Each entry focuses on a key writing feature or strategy, illustrates how it com- monly goes wrong, teaches the grammatical underpinnings necessary to understand it and offers suggestions to wield it effectively. We encourage readers to share comments on or suggestions for this section on Twitter, using the hashtag: #how’syourwriting?

## Editing

Editing can be a fight; wrestling a meandering draft into a concise, publishable paper challenges even seasoned writers. Editing means modifying, shaping, and fine-tuning the raw materials of that imperfect draft into a strong final product. Done well, editing focuses and elevates your writing. But editing risks endless loops of revisions that can leave you wondering whether you are strengthening the work at all.

You need a plan – a systematic approach to editing. To ensure that your editing boosts the quality of your work, remember to attend to the three ‘S’s: story, structure, and style. Story deserves some attention before you write a single word, and structure and style considerations will naturally arise as you write your draft. Too much in-the-moment editing, however, can stifle your writing momentum. Draft something first, then use this approach to ensure you’ve said what you really want to say.

## Edit at the level of the paper: think *story*

Your paper must tell a persuasive and compelling story. When editing for story, think about your target journal and its likely readers. Who are they, what do they care about, and what do they already know about the problem your study addresses? Typically, medical journals favour concise, pragmatic stories; their readers want to know how they can use research findings, or how those findings will shape their practice. Your story’s *hook* [1] – the ‘so what’ promise – needs to be addressed loud and clear, in both your introduction and your discussion.

Avoid the traps that derail good research stories. Although researchers embark on an intellectual journey in completing a study, readers need not be dragged through all the twists and blind alleys of that journey. Tell your story coherently, not necessarily chronologically. Limit jargon; unless you intend to write solely for your field’s insiders, be kind to your readers and explain terms that may be unfamiliar. Beware of throwaway references to theory; ground your story in theory when it makes narrative sense to do so. And resist becoming too attached – to a paragraph, a sentence, a result, even a turn of phrase. Sometimes the best edits begin with the courage to try a revision that deletes something you like, but that you feel in your gut doesn’t belong.

## Edit at the level of the paragraph: think *structure*

Good research stories thrive on a logical flow of ideas. Paragraphs are the structural foundation of any paper, and their arrangement and composition dictates how readily readers will be able to follow your logic. Each paragraph should be a coherent unit, addressing one topic. Start each paragraph with a topic sentence that flags what is to come for readers, and then ensure that the paragraph is true to the spirit of that sentence. Think about transitions between paragraphs, and compose topic sentences to show readers how what has gone before relates to what will come next. Consider this transitional topic sentence:

Although the procedures for carrying out grounded theory research are highly structured, the criteria for evaluating the quality of a grounded theory study are less clear.

This sentence signals a shift in topic, from the procedural focus of the previous paragraph to the evaluative focus of the new paragraph. A struggle to link paragraphs convincingly can be a symptom of faulty composition. Consider, when transitions prove challenging, whether paragraphs are in the wrong place, or whether one paragraph doesn’t belong at all.

## Edit at the level of the sentence: think *style*

Sentence-level editing should make the incorrect correct – fixing grammatical errors – but it should also aim to make the correct better by attending to style. Effective sentence-level editing requires us to recognize when sentences are awkward or unclear, to identify the source of that awkwardness, and to deploy a range of remedies. Entire books promise to build these critical skills so that writers can inject polish and style into their writing. Here, I offer only three key pieces of advice:

## Power up your verbs

The inaugural Writer’s Craft addressed verbs – no accident, as verbs are the engines of our stories [2]. Successful editing replaces weak verbs with stronger ones. Forms of the verb ‘to be’ (is, are, was, were, be been) often underperform in sentences, and cry out for strengthening.

For example:

Stress **is **a frequent problem facing medical students

might be re-crafted as …

Stress **plagues** medical students

for greater impact.

## Prune needless words

Identify and eliminate unnecessary words and redundant phrases. Pinker has compiled helpful lists of what he calls ‘morbidly obese phrases’ [3]; writers would do well to affix these to their computer screens while they edit. Some of these phrases can be replaced with leaner equivalents; for example, substituting ‘if’ for ‘in the event that’ or ‘we must’ for ‘it is imperative that we.’ Other bloated phrases can simply be eliminated altogether; ‘it is a well-known fact that’ need never start a sentence, for example.

Constructions involving ‘it’, ‘this’, ‘that’, and ‘there’ invite our editorial attention also; these constructions contribute to what Helen Sword calls ‘flabby prose’ [4].

Instead of …

There are many faculty members who are frustrated by the limited time that they have available for teaching.

Consider …

Lack of time frustrates many faculty teachers.

Finally, train your editorial attention to adverbs. We can often render these verb modifiers obsolete by choosing stronger, more evocative verbs.

Instead of …

Educators **strongly recommend** feedback as an essential and indispensable element of clinical learning.

Consider …

Educators **champion** feedback’s indispensable role in clinical learning.

Note that I have not only substituted the verb ‘champion’ for the weaker phrase ‘strongly recommend’, but I have also eliminated the word ‘essential’, which is rendered redundant by the stronger ‘indispensable’.

## Limit nominalizations

Nominalizations are nouns formed from other parts of speech, especially verbs. Academic writing overflows with them; words like *contribution, participation, development, *and *indication* abound in journal articles. Buried in these familiar nouns are active verbs – *contribute, participate, develop, indicate *– whose power has been neutralized by reconstituting them as nouns. Sword calls nominalizations ‘zombie nouns’ [5], and their impact is to weigh down prose, sapping its energy and verve. When editing, root out these deadweights whenever possible.

Consider the difference between the following two sentences:

The learning process involves the initial **demonstration** of skills in a simulated setting, followed by the **application** of those skills to a real clinical situation.

Learners must first **demonstrate** skills in a simulated setting, then **apply** those skills to a real clinical situation.

A nominalization typically contains the verb you need to enliven your sentence; the editorial trick is to liberate it.

Editing requires writers to critically assess their own work as a prelude to improving it – no easy feat. I have found three productive strategies to address this challenge: 1) I read my work aloud, which helps me to think like a reader rather than a writer, 2) I allow a gap between writing the draft and editing it, which softens my attachment to words and phrases that might need to be discarded, and 3) I invite a few colleagues and friends to weigh in, supplementing my self-assessment with voices of reason. But while critical self-assessment is productive, perfectionism is not. Editing can continue indefinitely; much published work could have been improved further. At some point – for your own sanity – you must be able to say ‘good enough’.

At least until you receive the reviews.
